# A Table of the Proportionate Doses of Medicine, from One Grain to One Ounce, &c. for Every Different Age

**Published:** 1816-08

**Authors:** Richard Walker

**Affiliations:** Oxford


*To the Editors of the London Medical and Physical Journal.*

GENTLEMEN,

YOU did me the favour of inserting, at page 18 of the 185th Number of your Journal, a sketch or kind of prospectus of a newly-adjusted Table for apportioning the doses of medicines to different ages. Since that time, having fully completed my original design, I take the liberty of sending it to you, with the hope that it may not be unacceptable. I am, Gentlemen, your's, &c.

*Oxford; June* 10, 1816. RICHARD WALKER.

*A Table of the proportionate Doses of Medicine, from One Grain to One Ounce, &c. for every different Age.*

| years 35, 34 | gr. 1 | gr. 2 | gr. 4 | gr. 6 | gr. 8 | gr. 10 | ℈j. | ʒj. | ℥j. | ℔j. or ℥xvi. |
|---|---|---|---|---|---|---|---|---|---|---|
| 33, 32 | 79/80 | 1 9/20 | 3 9/10 | 5 7/8 | 7 7/8 | 9 4/5 | gr. 19 3/4 | gr. 59 | ʒ 7 4/5 | ℥ 15 4/5 |
| 31, 40—30 | 39/40 | 1 9/10 | 3 7/8 | 5 4/5 | 7 4/5 | 9 3/4 | 19 1/2 | 58 | 7 3/4 | 15 3/4 |
| 29 | 29/30 | 1 7/8 | 3 4/5 | 5 3/4 | 7 3/4 | 9 2/3 | 19 1/4 | 57 | 7 2/3 | 15 1/2 |
| 28 | 19/20 | 1 6/7 | 3 4/5 | 5 2/3 | 7 2/3 | 9 1/2 | 19 | 56 | 7 1/2 | 15 1/4 |
| 27 | 15/16 | 1 5/6 | 3 3/4 | 5 1/2 | 7 1/2 | 9 1/3 | 18 2/3 | 55 | 7 1/3 | 15 |
| 26 | 11/12 | 1 4/5 | 3 3/4 | 5 1/2 | 7 1/3 | 9 1/4 | 18 1/3 | 54 | 7 1/4 | 14 1/2 |
| 50—25 | 9/10 | 1 3/4 | 3 1/2 | 5 1/3 | 7 1/4 | 9 | 18 | 53 | 7 | 14 |
| 24 | 7/8 | 1 3/4 | 3 1/2 | 5 1/4 | 7 | 8 3/4 | 17 1/2 | 52 | 6 3/4 | 13 1/2 |
| 23 | 17/20 | 1 2/3 | 3 1/3 | 5 | 6 3/4 | 8 1/2 | 17 | 51 | 6 2/3 | 13 |
| 22 | 5/6 | 1 2/3 | 3 1/3 | 4 4/5 | 6 2/3 | 8 1/4 | 16 1/2 | 50 | 6 1/2 | 12 2/3 |
| 21 | 4/5 | 1 1/2 | 3 1/4 | 4 3/4 | 6 1/2 | 8 | 16 | 48 | 6 1/4 | 12 1/3 |
| 60—20 | 3/4 | 1 1/2 | 3 | 4 1/2 | 6 | 7 1/2 | 15 1/4 | 45 | 6 | 11 3/4 |
| 19 | 2/3 | 1 1/3 | 2 4/5 | 4 1/3 | 5 3/4 | 7 1/4 | 14 1/2 | 43 | 5 2/3 | 11 |
| 18 | 2/3 | 1 1/3 | 2 3/4 | 4 1/4 | 5 2/3 | 6 3/4 | 13 1/2 | 40 | 5 1/3 | 10 |
| 17 | 3/5 | 1 1/4 | 2 1/2 | 4 1/4 | 5 1/3 | 6 1/4 | 12 1/2 | 38 | 5 | 9 1/2 |
| 70—16 | 3/5 | 1 1/6 | 2 1/3 | 4 | 5 | 5 3/4 | 11 1/2 | 35 | 4 2/3 | 9 |
| 15 | 4/7 | 1 1/8 | 2 1/4 | 3 1/2 | 4 1/2 | 5 1/2 | 10 3/4 | 32 | 4 1/3 | 8 1/2 |
| 14 | 1/2 | 1 | 2 | 3 | 4 | 5 | 10 | 30 | 4 | 8 |
| 80—13 | 3/7 | 6/7 | 1 4/5 | 2 3/4 | 3 2/3 | 4 1/2 | 9 1/4 | 28 | 3 2/3 | 7 1/4 |
| 12 | 5/12 | 5/6 | 1 3/4 | 2 1/2 | 3 1/3 | 4 1/4 | 8 1/2 | 25 | 3 1/3 | 6 1/2 |
| 90—11 | 3/8 | 3/4 | 1 1/2 | 2 1/4 | 3 | 3 3/4 | 7 3/4 | 23 | 3 | 6 |
| 10 | 1/3 | 2/3 | 1 1/3 | 2 | 2 3/4 | 3 1/2 | 7 | 21 | 2 3/4 | 5 1/2 |
| 100— 9 | 1/3 | 2/3 | 1 1/4 | 1 2/3 | 2 1/2 | 3 | 6 1/4 | 18 | 2 1/2 | 5 |
| 8 | 1/4 | 1/2 | 1 1/8 | 1 1/2 | 2 1/4 | 2 3/4 | 5 3/4 | 17 | 2 1/4 | 4 1/2 |
| 7 | 1/4 | 1/2 | 1 | 1 1/3 | 2 | 2 1/2 | 5 | 15 | 2 | 4 |
| 6 | 1/5 | 3/8 | 7/8 | 1 1/4 | 1 3/4 | 2 1/4 | 4 1/2 | 13 | 1 3/4 | 3 1/2 |
| 5 | 1/6 | 3/8 | 3/4 | 1 1/4 | 1 1/2 | 2 | 4 | 12 | 1 2/3 | 3 |
| 4 | 1/7 | 1/3 | 2/3 | 1 | 1 1/3 | 1 3/4 | 3 1/2 | 10 | 1 1/3 | 2 1/2 |
| 3 | 1/8 | 1/4 | 1/2 | 4/5 | 1 1/4 | 1 1/2 | 2 3/4 | 8 | gr. 64 | 2 |
| 2 | 1/10 | 1/5 | 2/5 | 3/4 | 4/5 | 1 | 2 1/4 | 7 | 56 | 1 3/4 |
| 1 | 1/12 | 1/6 | 1/3 | 2/3 | 3/4 | 4/5 | 1 2/3 | 5 | 40 | 1 1/2 |
| months 9 | 1/14 | 1/7 | 1/4 | 1/2 | 2/3 | 3/4 | 1 1/3 | 4 | 32 | 1 |
| 6 | 1/20 | 1/10 | 1/5 | 1/4 | 2/5 | 1/2 | 1 | 3 | 24 | ʒ 6 |
| 3 | 1/30 | 1/15 | 1/8 | 1/6 | 1/4 | 3/8 | 3/4 | 2 | 16 | 4 1/2 |
| 1 | 1/36 | 1/18 | 1/9 | 1/7 | 1/5 | 1/3 | 2/3 | 1 2/3 | 14 | 4 |
| weeks 3 | 1/45 | 1/22 | 1/12 | 1/8 | 1/6 | 1/4 | 1/2 | 1 1/2 | 12 | 3 |
| 2 | 1/60 | 1/30 | 1/15 | 1/11 | 1/7 | 1/6 | 1/3 | 1 | 8 | 2 |
| 1 | 1/80 | 1/40 | 1/20 | 1/15 | 1/10 | 1/8 | 1/4 | 3/4 | 6 | 1 2/3 |
| days 4 | 1/100 | 1/50 | 1/25 | 1/18 | 1/12 | 1/10 | 1/5 | 3/5 | 5 | 1 1/3 |
| 2 | 1/120 | 1/60 | 1/30 | 1/22 | 1/15 | 1/12 | 1/6 | 2/3 | 4 | 1 |
| 0 | 1/144 | 1/72 | 1/36 | 1/27 | 1/18 | 1/14 | 1/7 | 3/7 | 3 | 2/3 |

*Observations.*

*Observations.*—In this Table, the column of ℈j. is the radical column, which is itself adjusted, as nearly as is necessary, to the different ages throughout; and all the other columns are adjusted to this, as nearly as is necessary and convenient for practice, neither of them being mathematically correct in all their points, such accuracy here being deemed both inconvenient and useless.

The ℔j. or ℥xvi., forming the last column of quantity, signifies the liquid pint in measure; and is applicable to the quantities or doses of blood to be taken away, and to the diurnal quantity, taken in convenient portions, of diet drinks, wine, &c.

In this Table, the age of 35, not 21, is considered and assumed as the acme of bodily strength or vigour, and requiring, *cæteris paribus*, the largest or full dose of any medicine.

The Table consists of two grand divisions, the first from 35 years to 1 year inclusively, which comprehends twelve parts; and from 1 year, inclusively, to birth, which includes another twelve parts, making, in the whole, exactly 144 parts; and the subdivisions are, not according to an uniform ratio of increasing age, but according to the actual increments of bodily strength, in the course of increasing age.

This Table must be considered as perfect in itself; but, in order to accommodate it to the Table of *Gaubius*, the one hitherto in ordinary use, where the full dose, as it is called, is assumed at 21, the following allowance must be made:—add one-fourth part more of the quantity given in this Table, at every age, throughout; thus, assuming or administering 5 grains for 4, 20 grains for 16, 25 for 20, &c.

It must be recollected, that, although the age of 21, the adult age, as it is called, or the age of full growth, respecting stature, is, in the table of Gaubius, assumed as the age for the full dose, yet it is well known, that, after this age, the bodily strength continues increasing, requiring an increased dose of any medicine, which is, and must be attended to, but is ordinarily adapted in a very vague or indefinite manner.

In the column of ages, 0 at the bottom, or commencement of the scale, signifies, of course, earliest infancy. Infants, especially whilst under a year old, vary very much, respecting strength of constitution, at different ages: the doses for such, are, therefore, fixed with particular precaution, and may frequently require to be somewhat increased.

The doses are regulated according to an ordinary habit of body, respecting strength; therefore the dose may be somewhat what increased, or diminished, as circumstances may require—for females, a deduction being made, according to circumstances, of about one-fifth or one-fourth part.

It will be apparent, that the construction of this Table demanded a degree of minuteness which may be occasionally dispensed with; it will oftentimes be sufficient in practice to take the nearest integral number, or nearest simple fraction.

The proportionate doses, throughout, apply to liquids as well as solid substances, recollecting that one minim in measure is equal to one grain in weight; and that in spirituous medicines, as tinctures, &c. two drops, ordinarily speaking, are equal to one minim; consequently, that, though one grain and one minim are each the sixtieth part of a drachm, 120 drops of any spirit, or tincture, ordinarily speaking, make one drachm in measure.

The intermediate or omitted quantities, in the doses, at the top of the columns, may be supplied, with little trouble, by dividing, doubling, or combining those which are given, which may be easily done, so as to produce, extemporaneously, any required quantity, or a regular gradation from the grain, or lower, to the ounce, or higher.

Where very minute portions of an article are required, whether liquid or solid, for division of doses, the following method may be adopted: mix any given portion of that article, intimately, with any conveniently larger portion of another article, used merely as a vehicle, and then divide the mixture into the required doses; thus, for instance, mix it so that a scruple, a drachm, or an ounce of the compound shall contain the required full dose of the medicine itself; then referring to the corresponding column in the Table of Doses, the portion to be given of such compound will be found for any age required; thus, mixing one grain of opium in powder with nineteen grains of liquorice powder, the compound containing one grain of the medicine in the scruple, or twenty grains, the dose at every age, considering the scruple in that instance the full dose, may be found in the column of one scruple; adjusting, as convenience may point out, the quantity of the medicinal article to that of the article used merely or chiefly as a vehicle, in liquids as well as solids.

N.B.—As the doses in this Table are adjusted from 0 upwards in a uniformly progressive manner, if it should happen that a first dose be found too little, the dose at the line above may be taken; and, if too great, the dose at the line below. This rule applies more particularly to children. In some instances, stances, a like quantity is given in the Table for two ages together, the difference being too minute to require attention: in this instance, likewise, according to circumstances, the same rule may be observed.

It might be urged, perhaps, that I have descended, particularly in the upper part of the Table, to a degree of minuteness of division wholly unnecessary. I considered, however, that the Table would be incomplete by any omission; and, moreover, it serves to mark, in a philosophical view, in the first column of doses, a true and regular gradation of increasing strength in fractional parts, from birth up to the integral number at the complete acme of bodily vigour.

With respect to the merits or utility of this Table, I shall briefly remark, in addition to what I have observed before, that the utility and convenience of a standard Table of this nature is unquestionable. With regard to the various Tables hitherto in use, which are founded principally upon that of Gaubius, I think they have all objectionable points, each of which are obviated or supplied in the present Table: first, in assuming the age of 21 as the acme of bodily vigour or strength; secondly, in giving a uniform mechanical ratio, in the diminution of the doses, from the adult age, as it is considered, viz. 21, to infancy, disregarding the difference in the acquisition of bodily vigour or strength, at the different periods of age; and, thirdly, in being too brief or concise in its scale for practical use.

The construction of the present Table, which is the result of long experience, with much attention to these points, presented itself to me in consequence of the imperfections in that of Gaubius I have just pointed out, and the very great utility, or rather necessity, of a more perfect one.

In order to show the propriety, or rather necessity, of introducing a newly-adjusted table of doses, it will be sufficient to notice, that, according to that of Gaubius, upon which all others are fabricated, ten drops of tincture of opium, and half an ounce of Epsom salts, viz. the half of the dose for the adult age, or 21, are assigned to the age of 7! hence it happens that such a table of doses is almost wholly neglected by practitioners of experience, and tends to mislead the inexperienced.

I have selected and placed at the tops of the columns those full doses which are most in common use, and likewise such as will admit, as I have before intimated, by the means of dividing, doubling, or combining quantities, of readily furnishing any other quantity which might be required; moreover any person may, taking this table as his standard, extend or contract it, according to his wish.

 The

The column of ℈j. in this table being considered as the primary or fundamental column, and approaching, upon the whole, the nearest to mathematical exactness, will, attending to the fractional parts, by the ordinary rules of multiplication and division, produce any other apportionate dose which may be required.

In this table is given, likewise, the proportionate doses for declining bodily vigour or strength, by increase of years from 35 to 100, having myself visited a person reputed to be 105.

There is an anomaly respecting the adjustment of doses, for which no table can provide, nor indeed is any practitioner, *à priori*, prepared for, viz. a peculiar idiosyncrasy in the habit. For instance, I know one gentleman to whom one grain of calomel is an appropriate dose; and another, about the same age, who requires eight grains of the same medicine to produce an equal effect. These, however, are rare instances, and being previously known to the individual himself, the practitioner is, by him, apprized of it.

Habit, as is well known, influences the doses of medicines materially, particularly respecting cordials, opiates, and wine: this is a circumstance, of course, for which no table can be expected to provide.

I might have placed, conformably to former tables, the ordinary full dose, as it is called, at the age of 21, and extended the scale proportionably upwards; in general practice, however, where the variations in the ordinary full dose, arising from the various powers of different medicines, are almost infinite, little advantage, respecting convenience, would have been obtained, and it would have been accommodating a perfect table, as I consider this to be, to an imperfect one.

I beg leave to observe, that the construction of this Table, together with the directions respecting it, being intended by me as a standard table, has cost me much laborious attention, and, I trust, it will be found to merit adequate attention from the faculty.

