# Erratum

**Published:** 1817-03

**Authors:** 


Erratum.—In my "Table of proportionate Doses of Medicines, &c." in the Journal for August 1815, page 104, for $1\frac{9}{20}$ in the second line of the third column, read $1\frac{19}{20}$.

